# Special Anatomy and Histology

**Published:** 1844-07

**Authors:** 


### ART. XX.

*Special Anatomy and Histology.* By WILLIAM E. HORNER, Professor of Anatomy in the University of Pennsylvania, &c. Sixth Edition. —*Philadelphia*, 1843. Two Volumes, 8vo, pp. 536, 548.

PROFESSOR HORNER is one of the few of the anatomists of America whose names are well known in Europe. His discoveries or improved descriptions of the tensor tarsi muscle, and of the longitudinal fibres of the rectum which turn under the lower margin of its internal sphincter, have gained him a deserved reputation for special investigations; and the work before us shows that he is well versed in the general knowledge of anatomy and physiology. The present is the sixth large edition published since 1826; and it is enlarged beyond all its predecessors, by the addition of extensive extracts, from the General Anatomy of Henle (translated through the medium of the Encyclopédie Anatomique), and other modern writers. It has the advantageous form of a union of descriptive anatomy with general anatomy, and a good deal of physiology; a form so agreeable, that we almost regret that the extent to which the sciences have been separately cultivated in England, should make it now almost impossible to combine them in one text-book.

Although we have not thoroughly *worked through* both these volumes, yet we have examined them sufficiently to say that the descriptive anatomy is, on the whole, accurate, and is clearly written in the American variety of our language; the general anatomy has most of the merits of the sources from which it is derived, though written with that kind of uncertainty which might be expected from one not practised in the actual investigation of the subject; and the physiology is chiefly of the old and easy kind. In none of its parts would the work be sufficiently minute for our more advanced students; yet, for a beginner, we think there is not, in the text-books of this country, one so good: it nearly accomplishes that which, for junior students, would be perfect, if it were possible, in a modernized edition of Bell's Anatomy.

The only parts that would interest our readers are the few original observations inserted here and there by the author, and of which some are annexed. Speaking of the form of the head, he quotes a recent letter from a missionary, the Rev. Mr. De Smet, who has spent some years among the Indians, on the west side of the Rocky Mountains.

"The process of flattening the head exists among many of the tribes on the Columbia river. Among the Indians at the Cascades, and the Tschenouks at Fort-van Couver, I remarked several babes who were undergoing the barbarous process. They attach them to boards of about two feet in length, this sort of cradle is covered with a skin, with the hair outside; the child is stretched on it; its arms are tied close to the body with soft leather bandages; another skin is fastened to each extremity of the board and covers the child. A smooth strip of cedar bark, or of some other elastic wood, four or five inches broad, is fastened over the forehead of the babe, so tight that the eyes appear to start from their sockets. In this painful situation, I was told, they leave them for about a year; after which, the head has taken the form they wish to give it, and which they consider as a mark of distinction and of great beauty......The deformity disappears partly as they grow old......The Cascade Indians and Tschenouks are remarkable for their ingenuity in constructing convenient and beautiful canoes, nets, and wooden utensils; they are, in nowise, considered inferior to their round-head neighbours. Their constant intercourse with the whites has rendered them more vicious, poor, and indolent; they are much addicted to lying, stealing, and immorality." (Vol. i, p. 130.)

In the account of structure of tendons, the author says that he once applied the mode of treating ununited fractures by seton, to a case of rupture of the tendo Achilles, which, a long time after the accident, appeared unlikely to unite in the usual way. A seton of silk riband was introduced, and kept in its place for six weeks and a half: it produced considerable inflammation, but was followed by a perfect reunion of the ruptured ends of the tendon.

A case is mentioned (vol. ii, p. 278), in which the inferior cava, instead of passing at once into the right auricle, ascended on the dorsal vertebræ, in the place of the vena azygos, and, curving over the root of the right lung, united with the lower part of the superior cava. Another case is described (vol. i, p. 389), of a muscular male black subject, in whom the pectoralis minor and the sternal portion of the pectoralis major were absent; and another (vol. i, p. 432), in which the pronator carpi quadratus consisted of two triangular pieces, the bases of which were reversed.

